# Analysis of Chemisorbed Tribo-Film for Ceramic-on-Ceramic Hip Joint Prostheses by Raman Spectroscopy

**DOI:** 10.3390/jfb12020029

**Published:** 2021-05-01

**Authors:** Risha Rufaqua, Martin Vrbka, Dušan Hemzal, Dipankar Choudhury, David Rebenda, Ivan Křupka, Martin Hartl

**Affiliations:** 1 Faculty of Mechanical Engineering, Brno University of Technology, Technická 2896/2, 616 69 Brno, Czech Republic; Martin.Vrbka@vut.cz (M.V.); David.Rebenda@vut.cz (D.R.); krupka@fme.vutbr.cz (I.K.); Martin.Hartl@vut.cz (M.H.); 2 Department of Condensed Matter Physics, Faculty of Science, Masaryk University, Kotlářská 267/2, 611 37 Brno, Czech Republic; hemzal@physics.muni.cz; 3 Nano Mechanics and Tribology Laboratory, Department of Mechanical Engineering, University of Arkansas, Fayetteville, AR 72701, USA; dc020@uark.edu

**Keywords:** synovial fluid, film formation, Raman spectroscopy, bio-tribology, tribo-chemistry

## Abstract

To understand the possible lubricant mechanism in ceramic-on-ceramic hip joint prostheses, biochemical reactions of the synovial fluid and the corresponding frictional coefficients were studied. The experiments were performed in a hip joint simulator using the ball-on-cup configuration with balls and cups made from two types of ceramics, BIOLOX^®^forte and BIOLOX^®^delta. Different lubricants, namely albumin, *γ*-globulin, hyaluronic acid and three model synovial fluids, were studied in the experiments and Raman spectroscopy was used to analyze the biochemical responses of these lubricants at the interface. BIOLOX^®^delta surface was found less reactive to proteins and model fluid lubricants. In contrast, BIOLOX^®^forte ball surface has shown chemisorption with both proteins, hyaluronic acid and model fluids imitating total joint replacement and osteoarthritic joint. There was no direct correlation between the measured frictional coefficient and the observed chemical reactions. In summary, the study reveals chemistry of lubricant film formation on ceramic hip implant surfaces with various model synovial fluids and their components.

## 1. Introduction

A healthy synovial joint is essential for a comfortable and active function of the musculoskeletal system [1]. Skeletal organs consist of multiple tissues and structures that allow for smooth movement of the synovial joints [2]. Nevertheless, millions of people in the whole world suffer from bone and joint degenerative and inflammatory problems [3]. To improve life quality of these patients, hip joint arthroplasty is an available surgery, proficient to significant extent [4]. During this surgery, elements from orthopedic biomaterials are implanted within the human body [3]. Consequently, aseptic loosening accompanied by osteolysis is considered the primary reason for defection of total hip arthroplasty, causing instability and infection [5]. Substantial service-life of the implants is thus requisite to prevent their untimely failure [6].

Conventional hip prostheses can be categorized according to the material combination used for the femoral head on acetabular cup. The most common combinations are metal-on-polyethylene, metal-on-metal, ceramic-on-ceramic and ceramic-on-polymer prostheses; each design has its own specific risk-benefit profile when considering anatomic situation of the patient. Aseptic loosening is much less frequent in patients with ceramic-on-ceramic or metal-on-metal prostheses as metal-on-polymer is less suitable for this purpose due to an inflammatory reaction against polymer wear debris. However, increased metal ion concentrations in the blood are considered a risk factor due to metal allergies or hypersensitivity, especially for patients with renal insufficiency or in women of childbearing age, in which cases ceramic implants could be a better choice. In comparison with metal femoral heads, ceramic heads provide higher smoothness, resulting in lower friction coefficients, yet lower toughness, subsequent in the risk of fragile fracture when using ceramic-on-ceramic implant material [4,7]. Ceramic on ceramic bearings for implants were introduced more than four decades ago. Since then, three generations of Biolox ceramics were explored with progression in density, grain size and purity. As a result, current alumina ceramic (introduced as BIOLOX^®^forte) and zirconia toughened alumina ceramic (BIOLOX^®^delta) can both be exposed to wider range of residual stress upon wear compared to the first generation of Biolox. Making an advancement to ceramic-on-ceramic couplings in total hip arthroplasty, alumina-zirconia composite was introduced to the market in 2000 [8]. Phase transformation of the zirconia-toughened alumina (ZTA) has been observed by fluorescence piezo-spectroscopy and X-ray diffraction; subject to residual stress state, the tetragonal zirconia (t-zirconia) transforms to monoclinic zirconia (m-zirconia), while monoclinic content and the residual stress were found linearly correlated [9]. It is validated that this transformation occurs under hydrothermal conditions as well. Comparing monoclinic content and surface roughness, worn areas of the retrieved heads show higher surface roughness than heads that have undergone testing in a hip simulation [10]. The wear was confirmed as the main reason for the in vivo phase transformation in zirconia, micro-Raman mapping of the fractured articulating surface found the tetragonal-to-monoclinic conversion involved extensively within the region [8]. The stress-induced tetragonal-to-monoclinic polymorphic transformation of zirconia results in high flexural strength and fracture toughness. These features occur because of the microscopic crack-tip shielding mechanism of ZTA [11]. Thus, Raman experiments demonstrate compositional enhancement of BIOLOX^®^delta [12]. To investigate the changes in material properties of BIOLOX^®^delta femoral heads, Raman spectroscopy was used as an effective technique [8,10,11,13].

Within the contact of a hip replacement, lubrication mechanisms and film formation depend on the composition of the synovial fluid (SF). Eventually, SF composition affects tribological performance of the contact couple, thus impairing the service-life of the implant [14]. The lubricant concentration is predicted to be modified by chemical variations occurring within the synovial joint environment due to injury or disease [15]. Natural SF contains proteins (albumin and *γ*-globulin) as lubricants. In albumin, the α-helix structure is present to large extent, whereas β-sheet structure prevails in *γ*-globulin. The boundary film is affected by change of the protein lubricants-a lubricant containing albumin provides lower friction than lubricant comprising only *γ*-globulin [16]. On the other hand, the major hydrodynamic nonprotein component of joint SF is Hyaluronan or hyaluronic acid (HA) [17]. High concentration of HA in SF is essential for normal joint function [17]; HA provides necessary lubrication for the joint, reducing friction of the moving bones and diminishing wear of the joint [18,19,20]. The viscous and elastic properties both depend on size, interactions and concentration of HA molecules in the fluid [17,20]. Furthermore, the molecular weight of HA is significantly related to its rheological properties [21]. Due to presence of several carboxyl and hydroxyl groups, HA can be chemically modified [17,22]. Under specific conditions, an acid-protein complex forms a protective gel of concentrated fluid compressed between the cartilage surfaces [21,23]. Under inflammatory conditions of arthritic diseases (such as osteoarthritis or rheumatoid arthritis), high molar mass HA is degraded by reactive oxygen species, although in case of OA patients, the percentage of proteins increases [19,20]. As a consequence, the viscosity of SF is reduced together with HA lubricant and leads to impaired joint movement and pain [17,19]. Another substantial component of human SF, besides serum albumin, *γ*-globulin and HA, are the phospholipids, which can have a considerable effect on the friction and wear properties of the joints [24]. The largely saturated phospholipids are present in a typical joint, increasing the surface activity [25]. The presence of phospholipids also directly affects the structure of the proteins and their function [26]. The detected concentration of HA and phospholipids in normal human SF is comparable to concentration of HA and DPPC (dipalmitoyl phosphatidylcholine) needed for optimal lubrication [27]. Improved lubrication conditions are also obtained by adding phospholipids to the *γ*-globulin based fluid. A combined effect of HA and phospholipids caused more accrued lubricant film in the case of a complex fluid [28].

The significance of lubrication lies in minimizing the friction between surfaces of the joint. In the case of boundary lubrication, friction force depends on the properties of the thin film formed on the surface of the solids, often biased by additives present in the lubricants. Dowson et al. [29] mention HA as an additive of SFs. Within synovial joint, SF components influence essentially the coefficient of friction, both before and after joint replacement. A comprehensive comparison of frictional behavior of articular cartilage with respect to speed and load was conducted by Furmann et al. [30], also contemplating the effect of SF composition. It was stated that no variations in the friction coefficient are displayed by protein-based solutions, while adding HA and phospholipids induced its change. It is also proclaimed that model SF produce thicker adsorbed films on alumina ceramic than on zirconia toughened alumina ceramic [14].

The above studies suggest that correlation between biochemical and mechanical processes during lubrication film formation within hip joint replacement on both types of ceramic implants should be considered and studied. The impact of protein lubricants, including phospholipids and HA, on friction and wear should be taken into account to acquire in vivo conditions [31]. The human SF differs considerably from calf serum, especially in average total protein concentration [32]. In addition, the structural changes of individual SF components within ceramic implants after joint replacement are rarely addressed. Consequently, for proper understanding of the joint replacement condition and further improvement of its durability, chemical changes of individual SF components and three model human SF fluids have been considered in our study. As Raman spectroscopy- surpasses conventional techniques in terms of facility, selectivity and stability, it is presumed most suitable to explain the function of the fluids and performance of the ceramic implant material [8,10,11,13]. Alumina ceramic implants were analyzed previously by this spectroscopic technique several times and practical bio diagnostics under presence of protein interactions has also been reported [33].

In summary, chemical analysis of SF and biochemical behavior of the materials present within joint replacement including the study of tribological and biological properties of the SF of a joint replacement could improve the concept of the whole process [34].

## 2. Materials and Methods

In the experiment the ball and cup sets were made from two types of ceramics. Alumina ceramic ball used was BIOLOX^®^forte from Zimmer-sulox (28 mm) (Zimmer GmbH, Sulzerallee 8, 8494 Winterthur, Switzerland), and the cup of the same material was used from Smith and Nephew (Smith and Nephew Inc., 1450E Brooks Rd, Memphis, TN 38116, USA). The other set of ball and cup had zirconia-toughened alumina ceramic BIOLOX^®^delta (28 mm) ball, and the cup was also made from the same material. In this set, both ball and cup were received from Zimmer (Zimmer GmbH, Sulzerallee 8, 8494 Winterthur, Switzerland).

The artificial hip joint simulator has a base frame with acetabular cup and swinging pendulum with femoral head. The pendulum is driven with an electronic motor, which enables the machine to maintain continuous motion in the flexion-extension plane. The simulator imitates artificial hip joint conditions, including real geometry, body temperature and load. It is also capable of measuring real-time velocity profile, average friction coefficients and viscous effect. This mechanism was utilized to visualize lubricating films between artificial head and cup in real geometry and to understand the effect of diameter, clearance, and material during in situ observation of lubricant film formation [35,36]. The pendulum hip joint simulator was also used to assess the coefficient of friction to evaluate the impact of surface texturing of ultra-high molecular weight polyethylene acetabular cup [37].

**Figure 1:**
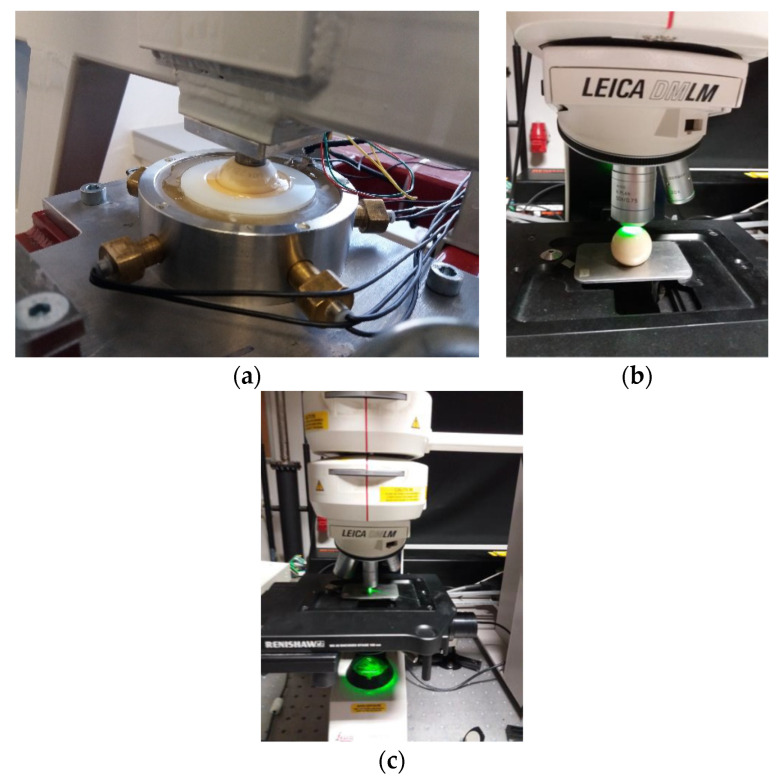
(**a**) Coefficient of friction measurement in the pendulum hip joint simulator. (**b**) Raman spectroscopic measurement of the ceramic ball. (**c**) Raman spectroscopic measurement of the lubricant in capillary.

The measurement of friction coefficient was operated with this novel pendulum hip joint simulator according to the method of evaluating viscous damping effect from the angular velocity profiles. Crisco et al. [38] used first this concept of evaluation of friction coefficient from a pendulum velocity profile. The cup is fixed within a stainless-steel pot using resin, the setup is supported by the base frame, as shown in Figure 1a. A rotating arm is linked to the head using a cone. With the beginning of the experiment, the pendulum arm is redirected to the primary position and then released and the flexion-extension swinging motion with a constant deflection lasted for 5 min. After that the pendulum drive was stopped, then only by a friction within contact the swinging motion was damped. As a result, via angular velocity sensor, instant deflection of the pendulum is recorded. The recorded signal is assessed using linear model of damping to obtain the friction coefficient and the measurement was derived from a curve of slowdown of pendulum oscillations. Therefore, angular velocity was calculated by the difference of maximum and minimum deflection to ascertain linear decay function. This method was used previously to determine friction coefficient [37,39,40,41]. The described instrumental setup allows the determination of the chemical reactions between the ceramic ball surface and SF, along with the frictional coefficient.

There are differences in content, fluid characteristics between Bovine Serum and human SFs. Thus, due to nonhuman origin, use of Bovine Serum to simulate joint replacement has been reprehended in recent times. To achieve more accurate lubrication that mimics human SF, three types of model SFs were used [24]. Using PBS, albumin, *γ*-globulin and HA solutions were produced as separate lubricants. For model SFs, PBS was used followed by addition of albumin, *γ*-globulin, HA and phospholipids. The components were dissolved in PBS overnight at 4 °C using laboratory rocker-shaker (MR-12, Biosan, Riga, Latvia). After that, each of the individual constituent solutions was mixed into one solution in order albumin, *γ*-globulin, HA, phospholipids. The specific products of the components used were Bovine serum albumin (powder, >96%; A2153, Sigma-Aldrich, St. Louis, MO, USA), *γ*-globulin from bovine blood (powder, >99%; G5009, Sigma-Aldrich, St. Louis, MO, USA), HA that is Sodium Hyaluronate HySilk (powder, quality class—cosmetic; molecular weight of 820–1020 kDa, Contipro, Dolní Dobrouč, Czech Republic) and phospholipids which is L-α-Phosphatidylcholine (powder, Type XVI-E, lyophilized powder; >99%; vesicles form; P3556, Sigma-Aldrich, St. Louis, MO, USA). Lubricant solutions were preserved at −22 °C after preparation. The types of prepared model SF represent healthy (or, physiologic), total joint replacement and osteoarthritic SF concentrations [24,30]. The concentration and combination of each of the lubricants used are described in Table 1.

The pendulum hip joint simulator experiment time was set up to 5 to 6 min to observe the tribological effect of each of the lubricants and obtain its chemical impact on the ball surface. The temperature was controlled at 37 °C. 532 N load was employed on the simulator during all experiments. The flexion-extension deviation range of the pendulum swinging was from −16° to +16°, velocity and rotation are linearly damped sinusoidal functions of frequency of 0.5 Hz. maximum Hertzian contact pressure was 29 MPa. After the tribological test, the balls and the lubricants were collected from the cup and preserved for 24 h at 4 °C before analysis.

Raman spectroscopy was found useful for determining the changes in the lubricants within the simulator. We used this methodology in our previous work [34] to explain the chemical reactions occurring within the artificial joint replacement. To acquire fingerprints of various lubricants before and after tribological experiments in the simulator, inVia Raman spectrometer by Renishaw was employed for analysis of the ball surfaces, as depicted in Figure 1b and lubricants as shown in Figure 1c using 532 nm excitation. The spectrometer was equipped with 1800/mm grating. The optical setup recorded spectra with step about 1/cm and the resolution of the equipment was more than 4/cm, as calibrated using Si reference. The excitation at 532 nm was provided by continuous DPSS laser, Genesis MX532 with tunable power by Coherent. To obtain Raman data of ceramic balls, 1 mW laser power was used on the surfaces with exposing time of 100 s. To observe the chemical structural changes of the lubricants before and after tribological tests, 100 mW laser power and 20 s exposition were applied. Due to the tribological process, differences in the chemical properties of the ball surfaces were observed.

## 3. Results and Discussion

### 3.1. Raman Analysis

**Figure 2:**
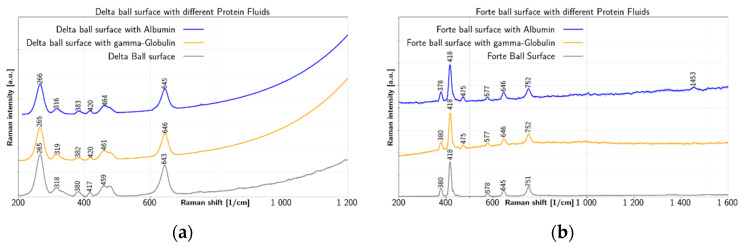
After-test Raman spectra of albumin and *γ*-globulin on (**a**) BIOLOX^®^delta and (**b**) BIOLOX^®^forte ball in comparison to clean balls.

Due to the compositional variability of the SF, the characteristics of formed films are dependent on the amount of present proteins and other constituents as well as physical conditions of the joint. The main objective of this research is to trace the chemical conditions present within the joint replacement, while measurement of frictional coefficient may also reveal some reality. Raman fingerprints of BIOLOX^®^delta and BIOLOX^®^forte hip implants after tribological test with albumin and *γ*-globulin are shown in Figure 2.

Concerning BIOLOX^®^delta ball surface, a prominent peak near 265 cm^−1^ is observed in Figure 2a both before and after tribological tests with albumin and *γ*-globulin. The marker bands of tetragonal zirconia are found at 265 cm^−1^, 318 cm^−1^, 459 cm^−1^ and 643 cm^−1^ [8]. Therefore, these bands are visible as basic skeleton of zirconia toughened alumina components, including the BIOLOX^®^delta. The second prominent peak of the clean BIOLOX^®^delta ball surface at 643 cm^−1^ shifts to 645 cm^−1^ after test with albumin and to 646 cm^−1^ after test with *γ*-globulin. Peaks at 318 cm^−1^, 380 cm^−1^, 417 cm^−1^ and 459 cm^−1^ visible in the spectrum of a clean BIOLOX^®^delta ball surface, shift to 316 cm^−1^, 383 cm^−1^, 420 cm^−1^ and 464 cm^−1^, respectively, after test with albumin and to 319 cm^−1^, 382 cm^−1^, 420 cm^−1^ and 461 cm^−1^, respectively, after test with *γ*-globulin. In contrast, the 380 cm^−1^ band is attributed to the monoclinic polymorph, while band at 419 cm^−1^ is described by Taddei et al. [13] as belonging to alumina. The latter, observed here for BIOLOX^®^delta ball without any tribological test at 417 cm^−1^, shifted to 420 cm^−1^ after test with both albumin and *γ*-globulin, which is agreement with value 419 cm^−1^, given by Taddei et al. [13]. In Figure 2b, the prominent peak of clean BIOLOX^®^forte surface at 418 cm^−1^ is unaffected by the tests. Similarly, the other peaks of the BIOLOX^®^forte surface at 380 cm^−1^, 578 cm^−1^, 645 cm^−1^ and 751 cm^−1^ show negligible shifts due to tests with albumin and *γ*-globulin. On the contrary, the peak at 475 cm^−1^ is visible on the BIOLOX^®^forte surface only after tests with albumin and *γ*-globulin. In addition, a small peak appears at 1453 cm^−1^ after test with albumin. Thus BIOLOX^®^forte surface exhibited before test a prominent peak at 418 cm^−1^ due to presence of alumina [13], this peak is also visible after tribological tests with both albumin and *γ*-globulin, without change of position. Other peaks recognizable for BIOLOX^®^forte components are found at 751 cm^−1^, 645 cm^−1^, 578 cm^−1^ and 380 cm^−1^ with only slight differences throughout all measurements. An exceptional peak at 1453 cm^−1^ is observed on BIOLOX^®^forte surface after test with albumin, providing information on CH_2_/CH_3_ deformation in the protein. In addition, a peak at 475 cm^−1^ is visible on the BIOLOX^®^forte surface with albumin and *γ*-globulin, which is due to C–C skeletal deformation [13,42,43]. Both peaks are probably markers of albumin and *γ*-globulin chemisorption on the surface of the BIOLOX^®^forte ball.

**Figure 3:**
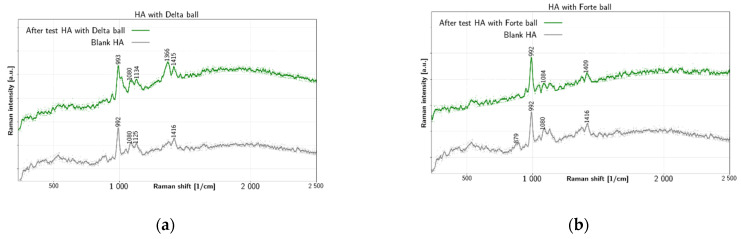
Raman spectra of HA liquid before and after test with (**a**) BIOLOX^®^delta and (**b**) BIOLOX^®^forte ball.

Figure 3 summarizes results of tribological tests for liquid HA. The HA fluid shows several peaks before test. There is a double peak near 1366 cm^−1^ and 1416 cm^−1^, which is due to C–H bending [44] and due to C–N stretching and C–H deformation, respectively. Additionally, there is a peak at 1080 cm^−1^ due to C–OH bending and acetyl group [44,45]. The prominent peak at 992 cm^−1^ is due to ring breathing vibration [46] (p. 482). The 879 cm^−1^ peak is also reported in literature [44] for HA. In Figure 3a HA collected after the tribological test with BIOLOX^®^delta shows change of the chemical structure, especially near 1000 cm^−1^: the ring-breathing mode at 993 cm^−1^ is altered and the C–OH bending peak remain at 1080 cm^−1^. In addition, the peak near 879 cm^−1^ is lost completely. In contrast, after test spectra with BIOLOX^®^forte in Figure 3b preserve the prominent HA liquid peak at 992 cm^−1^ together with most other peaks. The main features of HA fluid remain mostly unchanged after test with BIOLOX^®^forte. The most significant change is the shift of C-N stretching peak from 1416 cm^−1^ to 1409 cm^−1^ and dissapearance of the 879 cm^−1^ band. The main Raman markers of HA at 1047 cm^−1^, 1372 cm^−1^ and 1406 cm^−1^ are connected with C–C and C–O stretching, C–H bending and combination of C–N stretching and C–H deformation, respectively [44]. Other reported Raman bands of HA include 446 cm^−1^, 949 cm^−1^ and 2904 cm^−1^.

**Figure 4:**
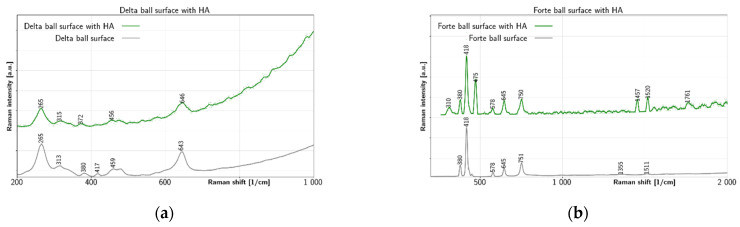
Raman spectra of ceramic surface after test with HA: (**a**) HA on BIOLOX^®^delta and (**b**) HA on BIOLOX^®^forte.

Figure 4 summarizes changes on the surface of balls after tests with HA. In Figure 4a, the prominent BIOLOX^®^delta ball surface peak at 265 cm^−1^ is unaffected by the test with HA. The second prominent peak at 643 cm^−1^ before test is shifted to 646 cm^−1^ after test with HA. The remaining BIOLOX^®^delta ball surface peaks at 313 cm^−1^, 380 cm^−1^, 417 cm^−1^ and 459 cm^−1^ are shifted a bit and become weaker with the test with HA. Thus, only tetragonal zirconia peaks at 265 cm^−1^ and 646 cm^−1^ remain well resolved. The other tetragonal zirconia peaks as well the alumina peak at 417 cm^−1^ are masked by strong after-test luminescence. Even though shifting is observed at 315 cm^−1^, 372 cm^−1^ and 456 cm^−1^ positions for the spectra of BIOLOX^®^delta surface after test with HA. Thus, for the BIOLOX^®^delta ball surface, some marker bands of tetragonal zirconia disappeared, and strong luminescence was observed after the experiment with HA. In Figure 4b all main peaks of the BIOLOX^®^forte surface are preserved after test with HA. In addition, there are several new after-test peaks with BIOLOX^®^forte: a strong peak at 475 cm^−1^, two peaks near 1500 cm^−1^ and two shoulders, at 310 cm^−1^ and at 1761 cm^−1^. The surface of BIOLOX^®^forte after testing with HA, luminescence was also observed, but in lesser extent and the peaks characteristic for the clean surface remain mostly unchanged, including the prominent 418 cm^−1^ alumina peak. More importantly, a peak on the after-test surface appeared at 475 cm^−1^ due to C-C skeletal deformation. This peak was also visible on the BIOLOX^®^forte surface after testing with albumin and *γ*-globulin. Further, well resolved peaks at 1457 cm^−1^, 1520 cm^−1^ and 1761 cm^−1^ appear after test with HA on the BIOLOX^®^forte surface, which cannot be identified with the clean surface peaks at 1355 cm^−1^ and 1511 cm^−1^. The peak at 1457 cm^−1^ gives information about CH_2_/CH_3_ deformation [46,47] (p. 480), while the 1761 cm^−1^ peak could be due to the C=O stretch [46] (p. 479). The peak at 1520 cm^−1^ and an additional peak at 310 cm^−1^ cannot be precisely defined. In summary, it can be assumed that chemical reaction took place between the ceramic balls and HA. For BIOLOX^®^forte, strong peak due to C-C skeletal deformation is found at 475 cm^−1^ after the test, this peak is also found for proteins on the same surface. In addition, four other peaks at 310 cm^−1^, 1457 cm^−1^, 1520 cm^−1^ and 1761 cm^−1^ are probably due to chemical adsorption of HA on the ball.

**Figure 5:**
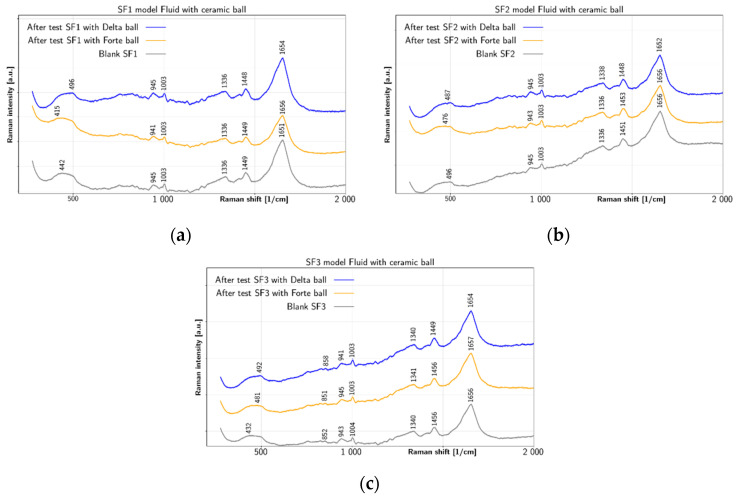
Liquid Raman spectra of model SFs before and after test: (**a**) SF1 with BIOLOX^®^delta and BIOLOX^®^forte and (**b**) SF2 with BIOLOX^®^delta and BIOLOX^®^forte (**c**) SF3 with BIOLOX^®^delta and BIOLOX^®^forte.

Figure 5 summarizes changes in model fluids SF1, SF2 and SF3 after tests with both types of ceramic balls. Apart from water peak at 1651 cm^−1^, the SF1 fluid shows in Figure 5a the before-test peaks at 1336 cm^−1^ and 1449 cm^−1^, together with smaller peaks at 945 cm^−1^ and 1003 cm^−1^ and a shoulder near 450 cm^−1^. All these peaks remain mostly unaffected by tests with ceramic balls. Therefore, model fluid SF1 has shown the smallest changes due to the test with the ceramic balls. The prominent peak near 1651 cm^−1^ before test is due to water, however, its shift to 1656 cm^−1^ with BIOLOX^®^forte ball and to 1654 cm^−1^ with BIOLOX^®^delta ball suggests contribution of the α-helix Amide I range (1645–1660 cm^−1^), which could be explained by bonding of albumin [48] (p. 217). Concerning other peaks, the 1449 cm^−1^ peak is an expression of CH_2_/CH_3_ deformation [46,47] (p. 480) and 1336 cm^−1^ peak comes from CH_2_–CH_3_ wagging [46,47] (p. 10). Two further peaks exhibited by all SF1 liquid spectra at 1003 cm^−1^ and 945 cm^−1^ are due to ring breathing [49] and C-C skeletal stretching α helix [49], respectively. The broad spectral feature shown in all SF1 spectra near 450 cm^−1^ could be due to C–C skeletal deformation [46] (p. 11). Similar to SF1, the before-test peaks of SF2 are shown in Figure 5b at 1336 cm^−1^, 1451 cm^−1^, 945 cm^−1^ and 1003 cm^−1^, including the shoulder near 490 cm^−1^. Even though the peaks at 945 cm^−1^, 1003 cm^−1^ and 1336 cm^−1^ remain mostly unaffected by the tests, the peak at 1451 cm^−1^ shifts to 1453 cm^−1^ after test with BIOLOX^®^forte ball, but to 1448 cm^−1^ after test with BIOLOX^®^delta ball. The spectra of SF2 liquid shows similar properties concerning the α-helix Amide I range, but there are also more visible changes. The SF2 CH_2_/CH_3_ deformation shifts from 1451 cm^−1^ before test to 1448 cm^−1^ after test with BIOLOX^®^delta and to 1453 cm^−1^ after test with BIOLOX^®^forte. The SF2 before-test 1336 cm^−1^ peak due to CH_2_–CH_3_ wagging shifts to 1338 cm^−1^ after test with BIOLOX^®^delta ball and cup pair. The results shown in Figure 5c for SF3 fluid are similar to the case of SF2. The main SF3 peaks before test are located at 1340 cm^−1^, 1456 cm^−1^, 943 cm^−1^ and 1004 cm^−1^, including the shoulder near 450 cm^−1^ and a small peak 852 cm^−1^. Most of these peaks remain unaffected by testing, but the 1456 cm^−1^ peak shifts to 1449 cm^−1^ after test with BIOLOX^®^delta ball. In addition, the peak at 943 cm^−1^ before test shifts to 945 cm^−1^ after test with BIOLOX^®^forte ball and to 941 cm^−1^ after test with BIOLOX^®^delta ball. In the spectra of SF3 liquid, the before-test water peak at 1656 cm^−1^ shifts after the test with both types of ceramic balls more significantly to 1654 cm^−1^ after test with BIOLOX^®^delta ball and cup. Since this peak lies in the range of α-helix Amide I band, the observed shifts may be due to changes in albumin, present in the SF3. The CH_2_/CH_3_ deformation peak is present at 1456 cm^−1^ in the before -test liquid and, similarly to SF2, it shifts more prominently (to 1449 cm^−1^) after test with BIOLOX^®^delta. The before-test values of the CH_2_–CH_3_ wagging peak at 1340 cm^−1^ and the ring-breathing peak at 1004 cm^−1^ remain mostly unaffected by the tests. On the other hand, the SF3 C–C skeletal stretching of α helix shifts from 943 cm^−1^ before testing to 941 cm^−1^ and 945 cm^−1^ after test with BIOLOX^®^delta and BIOLOX^®^forte ball and cup pairs, respectively. In addition, the C-C skeletal stretch shifts from 852 cm^−1^ without test to 858 cm^−1^ after test with BIOLOX^®^delta and to 851 cm^−1^ after test with BIOLOX^®^forte. The broad feature near 450 cm^−1^ in all SF3 fluid spectra is probably due to C-C skeletal deformation [46] (p. 11). Due to high Raman cross-section of phenyl group near 1000 cm^−1^ caused at visual wavelengths of excitation by its pre-resonance character due to the underlying delocalized p-electron system [50], tyrosine and tryptophan are good markers of protein presence, especially in case of resonant excitation [51]. Concerning specifically albumin, its Amide I and III bands are located near 1656 cm^−1^ and 1250 cm^−1^, respectively. In addition, intense bands near 1340 cm^−1^ due to sidechain stretching are observed [52]. In addition, albumin marker band near 940 cm^−1^ is also reported [53]. While in case of globulin the overall structure and the charge density profile of is related with electrostatic attraction between positive charges of protein and negative charges of polysaccharide. The hydrogen bonds are beneficial in case of the majority of carboxyl functions of polysaccharide are protonated [49].

**Figure 6:**
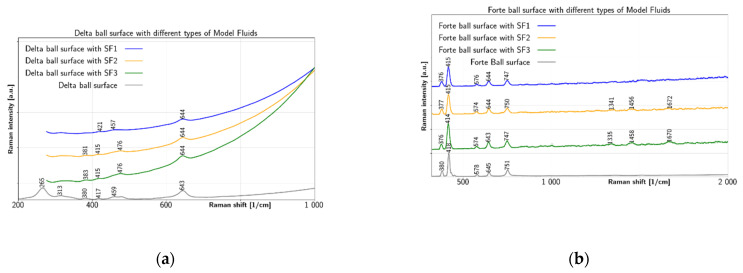
Comparison of Raman spectra on ceramic balls surfaces: (**a**) SF1, SF2 and SF3 on BIOLOX^®^delta and (**b**)SF1, SF2 and SF3 on BIOLOX^®^forte.

Figure 6a after testing with BIOLOX^®^delta ball, all three model fluids on the ball surface presented characteristic spectra found also on the clean BIOLOX^®^delta ball, only with slight peak shifts. The clean BIOLOX^®^delta surface provides peaks at 265 cm^−1^, 313 cm^−1^, 380 cm^−1^, 417 cm^−1^, 459 cm^−1^ and 643 cm^−1^. The peak at 380 cm^−1^ is diminished for SF1 and shifted to 381 cm^−1^ and 383 cm^−1^ for SF2 and SF3, respectively. The peak at 417 cm^−1^ is shifted to 421 cm^−1^ for SF1 while for the other two fluids it is left almost unaffected. However, after testing with SF2 and SF3, differences were found also on the BIOLOX^®^delta ball surface. Even though before-test peak at 459 cm^−1^ does not shift significantly after test with SF1, there is an apparent shift to 476 cm^−1^ after the test for both SF2 and SF3. The BIOLOX^®^forte ball surface with all three model fluids is shown in Figure 6b. The clean surface shows peaks at 380 cm^−1^, 418 cm^−1^, 578 cm^−1^, 645 cm^−1^ and 751 cm^−1^. The main peak at 418 cm^−1^ shifts near 415 cm^−1^ for all three fluids. The peak at 380 cm^−1^ downshifts near 376 cm^−1^ for all three fluids. The peak at 578 cm^−1^ shifts to 574 cm^−1^ for SF2 and SF3, but to 576 cm^−1^ for SF1. The peak at 751 cm^−1^ shifts to 747 cm^−1^ for SF1 and SF3, but to 750 cm^−1^ for SF2. The peak at 645 cm^−1^ seems to be almost unaffected by the fluids, but three weak peaks at 1341 cm^−1^, 1456 cm^−1^ and 1672 cm^−1^ for SF2 and 1335 cm^−1^, 1458 cm^−1^ and 1670 cm^−1^ appeared for SF3.Therefore, three types of model SFs conducted in the tribological tests with BIOLOX^®^delta and BIOLOX^®^forte hip implant balls mimicked the condition of a healthy joint (SF1), a total joint replacement (SF2) and an osteoarthritic joint (SF3). The distinctly smallest changes in the fluid were shown after tests by SF1, which had the lowest protein content in our study. One can thus conclude that elevated protein content is a significant factor for interaction with the ceramic balls of both types. Concerning the ball surfaces, BIOLOX^®^delta showed smaller differences after tests for SFs. Thus, for the tribological test of model SFs with BIOLOX^®^delta, it can be assumed that there is little reaction between proteins and the ball surface. This is in accordance with Parkes et al. [10], who state that phase transformation of the BIOLOX^®^delta femoral head was not triggered by wear simulated implants. On the other hand, the elevated after-test reaction of the BIOLOX^®^forte surface was confirmed also with individual SF protein constituents and HA. In particular, after testing with BIOLOX^®^delta ball, all three model fluids on the ball surface presented characteristic spectra found also on the clean BIOLOX^®^delta ball, only with slight peak shifts. However, after testing with SF2 and SF3, differences were found also on the BIOLOX^®^delta ball surface. Even though before-test peak at 459 cm^−1^ does not shift significantly after test with SF1, there is an apparent shift to 476 cm^−1^ after the test for both SF2 and SF3. In addition, the before-test peak at 380 cm^−1^ shifts to 381 cm^−1^ after test with SF2 and to 383 cm^−1^ after test with SF3. More pronounced changes after tests with SFs were provided by the BIOLOX^®^forte balls. Most significant is the appearance of new bands near 1350 cm^−1^, 1460 cm^−1^ and 1670 cm^−1^ after the tests with SF2 and SF3, confirming again the higher activity of these two fluid types. The 1460 cm^−1^ band lies in the region of CH_2_/CH_3_ deformation [46,47] (p. 480), a peak in this range was also found on the BIOLOX^®^forte surface while reacting with HA. The peak near 1670 cm^−1^ may give Amide I evidence of the β-sheet structure. The peak near 1350 cm^−1^ is likely due to CH_2_–CH_3_ wagging.

### 3.2. Microscopic View of the Formed Films

**Figure 7:**
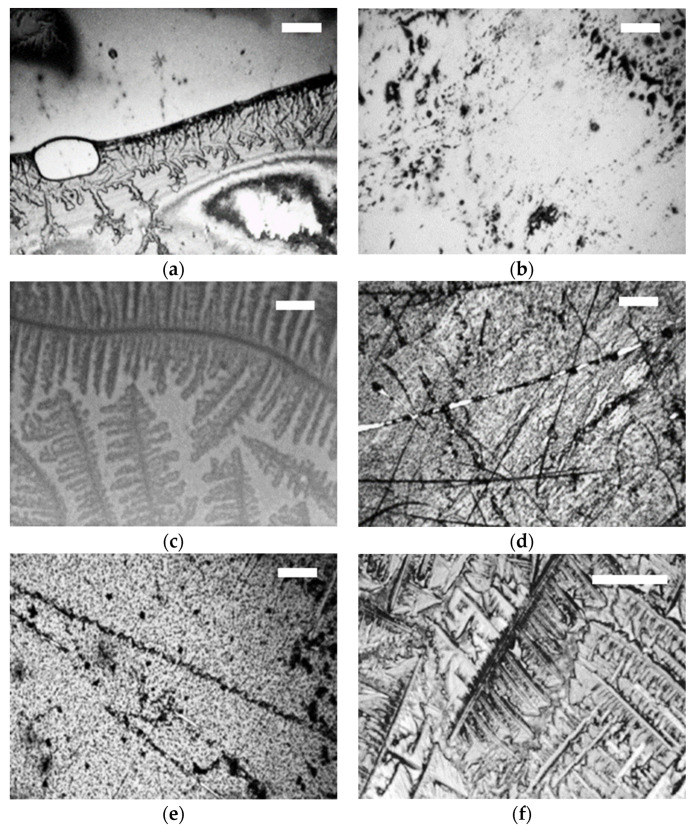
Optical images of BIOLOX^®^delta ball modified with (**a**) albumin, (**b**) *γ*-globulin, (**c**) HA and of BIOLOX^®^forte ball modified with (**d**) albumin, (**e**) *γ*-globulin and (**f**) HA. The scale bars are 100 µm for (**a**,**b**–**e**), 10 µm for (**c**) and 50 µm for (**f**).

In Figure 7, optical images of the ceramic balls surface after tribological test in the simulator are shown for individual SF components. Less crystallization was observed on ceramic balls for albumin and *γ*-globulin than in case of metal balls, as found in our previous work [34]. In the case of albumin solution on BIOLOX^®^delta, a flow of the solution was observed in Figure 7a. The rest of the balls Figure 7b–e show crystallites of the protein molecules. Concerning optical images, the protein fluids do not show any significant changes on the BIOLOX^®^delta ball surface. In the microscopic view of the BIOLOX^®^delta surface for both albumin and *γ*-globulin, a thin film was observed, with probably no chemical adsorption to the BIOLOX^®^delta surface. In contrast, in the case Figure 7c,f of HA on both BIOLOX^®^delta and BIOLOX^®^forte balls, a patterned film was distinguishable. As the two ceramic balls exhibit a differently patterned film in the microscopic picture for HA, it can be presumed that HA is adsorbing chemically on both balls, but different chemical structure is created by HA for the BIOLOX^®^forte and the BIOLOX^®^delta surfaces.

**Figure 8:**
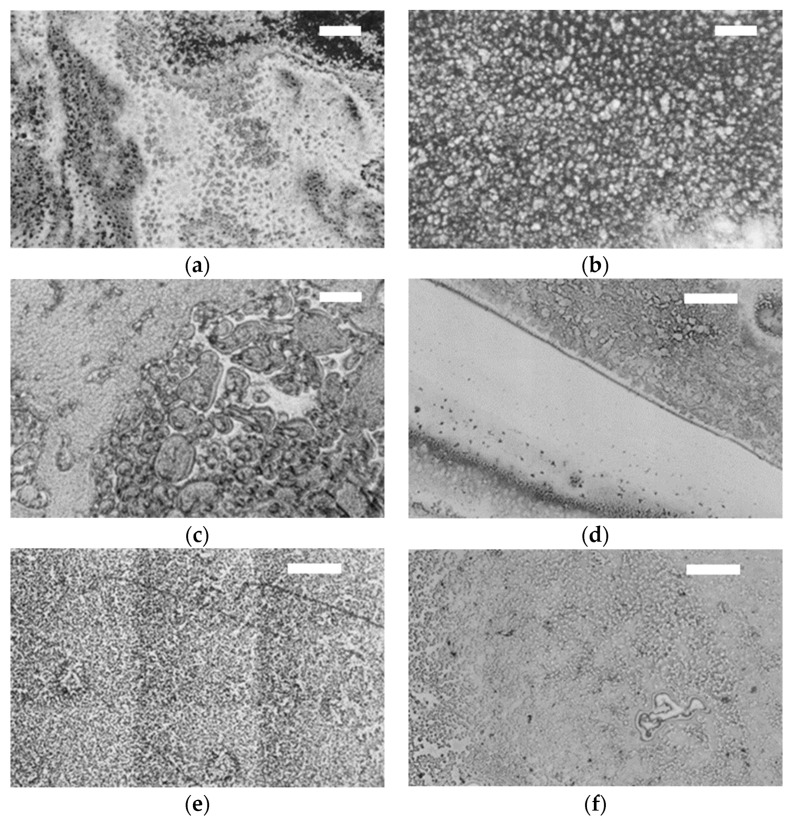
Optical images of BIOLOX^®^delta ball modified with (**a**) SF1, (**b**) SF2, (**c**) SF3 and of BIOLOX^®^forte ball modified with (**d**) SF1, (**e**) SF2 and (**f**) SF3. All scale bars are 100 µm.

Figure 8 shows the ceramic balls surface with model SFs after the tribological test. SF1 produced on both BIOLOX^®^delta and BIOLOX^®^forte balls Figure 8a, d, respectively, flows of the liquid across the ball surface. However, on BIOLOX^®^delta ball, the liquid was found almost on all surface, while on BIOLOX^®^forte ball, part of the surface remained almost without fluid as shown in Figure 8d. For SF2 on BIOLOX^®^delta and BIOLOX^®^forte ball surface Figure 8b,e, respectively, the model fluid spread on the whole surface of the balls more evenly compared to SF1. For SF3 on BIOLOX^®^delta ball surface Figure 8c, the film showed significant variation in concentration, while on the BIOLOX^®^forte ball surface Figure 8f the same fluid was spread more consistently. Concerning microscopic images of model SFs, the SF1, mimicking a healthy joint, produced uneven films on both ceramic balls. In addition, some parts of the BIOLOX^®^forte surface are clear without any visible film. On the contrary, the SF2, mimicking a total joint replacement, produced uniform distribution of fluid components on the ceramic ball surfaces. Finally, the SF3, mimicking an osteoarthritic joint, displayed inconsistent accumulation of fluid on the balls surface in the microscopic picture. Concerning film thickness, for ceramic implants a slightly increasing tendency of adsorbing layer was shown by the model fluids. Finally, a thin layer of the film was observed [14].

### 3.3. Coefficient of Friction Analysis

**Figure 9:**
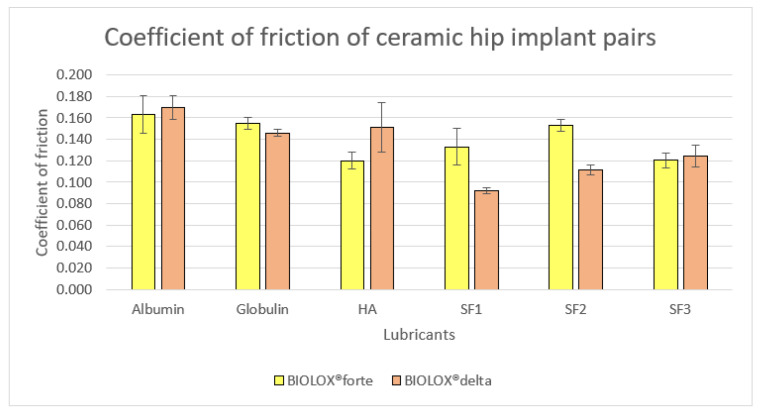
Coefficient of friction for BIOLOX^®^delta and BIOLOX^®^forte Ceramic contact pairs, respectively, for albumin, *γ*-globulin, HA and SF1, SF2 and SF3 model SFs.

Coefficient of friction was measured for both BIOLOX^®^delta and BIOLOX^®^forte ceramic ball and cup contact pairs with albumin, *γ*-globulin, HA and all three types of model fluids SF1, SF2 and SF3, each measurement were conducted three times. In Figure 9 the coefficient of friction values for both joint pairs with all six lubricants are described with statistical analysis. For albumin, BIOLOX^®^delta provides mean value 0.170 and BIOLOX^®^forte provides 0.163, which are very close. Accordingly, *γ*-globulin results in coefficient of friction with smaller variation between BIOLOX^®^delta and BIOLOX^®^forte surfaces, 0.0146 and 0.155, respectively. On the other hand, the value for HA with BIOLOX^®^forte ball, 0.120, is lower than the value 0.151 with BIOLOX^®^delta, the standard deviation for HA with BIOLOX^®^delta also showed comparatively higher. For the model fluids, values of coefficient of friction for BIOLOX^®^delta and BIOLOX^®^forte balls showed sufficient differences in case of SF1 and SF2, values for BIOLOX^®^forte are considerably higher than that for BIOLOX^®^delta. For SF3 with BIOLOX^®^delta contact pair, a bit higher mean value 0.125 is shown than for SF3 with BIOLOX^®^forte mean, 0.12. With BIOLOX^®^delta contact pair the coefficient of friction was found to be higher in the case of albumin, HA and a bit higher in case of osteoarthritic model SF compared to BIOLOX^®^forte contact pair. However, the BIOLOX^®^forte contact pair exhibited a higher mean coefficient of friction in the case of *γ*-globulin, model SF of healthy joint and model SF of total joint replacement. Literature shows that the friction coefficients are influenced more by the combination of materials specifically in case of ceramic-on-ceramic implants, than by the diameter of a femoral head [41]. Kasuka et al. [54] measured friction between flat surfaces of three ceramics, lubricated with bovine serum solution. Coefficient friction for alumina showed 0.05 at 35 °C. While for alumina nanocomposites friction measurement were carried out using a ball-on-plate tribometer, with distilled water and fetal bovine serum solution (FBSS). The coefficient of friction was around 0.3–0.5 for FBSS and approximately 0.4–0.7 for distilled water [55]. With 25% bovine serum friction coefficient was investigated for BIOLOX^®^forte (32 mm) using hip friction simulator, cup positioned with 0° and 75°abduction angle. Under 2500 N load friction coefficient was in the region of 0.2 with 0° abduction angle and at 75° abduction angle three specimens showed 0.12, 0.054 and 0.083, respectively [56]. While the friction coefficient derives from a curve of slowdown of pendulum oscillations, using the same pendulum hip joint simulator was studied with bovine serum lubricant and using the load 75 kg. The friction coefficient for ceramic-on-ceramic surfaces was measured 0.11 to 0.12 [41].

Albumin and *γ*-globulin showed almost no chemical reactivity for BIOLOX^®^delta ceramic, whereas chemisorption took place for BIOLOX^®^forte with albumin and *γ*-globulin both proteins individually. The coefficient of friction result for these two proteins with BIOLOX^®^delta and BIOLOX^®^forte contact pairs are very close values. On the contrary for SF1 and SF2, BIOLOX^®^delta and BIOLOX^®^forte contact pairs coefficient of friction showed vast differences, where BIOLOX^®^forte value was higher and SF3 showed very close mean values for both contact pairs. While chemisorption took place in case of BIOLOX^®^forte specifically for SF2 and SF3, but BIOLOX^®^delta showed almost no chemical reactivity for all three types of model fluids. On the other hand, HA was reactive for both ceramic contact pairs, even though between BIOLOX^®^delta and BIOLOX^®^forte mean values of friction are quite variable with HA.

Therefore, in accordance with the occurrence of chemisorption no linear relationship with the coefficient of friction observed. Hence the variation of coefficient of friction could not be correlated with the occurrence of chemical reaction within the ceramic contact pairs with model SFs and its components It can be concluded that frictional behavior of the contact pair is not an influencing factor of the chemisorption process of SF lubricant on the ceramic implant materials surfaces.

In the present study, chemical composition and chemical structure of the SF films are apprehended while differentiating the variability of the SF lubricants. However, four main constituents of SF albumin, *γ*-globulin, HA and phospholipids were considered here as lubricants while limiting the effect of other components present in SF in trace amount to entail simplification of the methodology. Nevertheless, we could determine the chemical reactivity of each of these components of SF separately and within a mixture with ceramic implant head. Additionally, it was possible to indicate the structural changes of lubricants due to tribological activity. In addition to the above chemical assignment of the observed vibrational bands, we also provide the observed peak widths (FWHM). In general, all peaks of the SF constituents are broadened at the BIOLOX^®^delta ball surface, but we attribute this behavior to broader peaks in the spectrum of the ball itself, compared to BIOLOX^®^forte ball. This conclusion is supported by observation of after-test liquids without presence of the ball in the measurement, when the peaks of all test SF become comparable. As expected, the narrowest peak comes from the aromatic breathing mode of the phenyl group. For brevity, the data are summarized in Appendix A (Table A1, Table A2, Table A3, Table A4 and Table A5).

## 4. Conclusions

Using Raman spectroscopy, this study analyzed chemisorption of individual components of the SF (albumin, *γ*-globulin and HA) and three model SFs to the hip joint ceramic balls.

There was no significant reaction between BIOLOX^®^delta and the proteins. Neither albumin nor *γ*-globulin adsorbed chemically on the BIOLOX^®^delta ball surface. In contrast, the chemisorbed films of both proteins were identified on BIOLOX^®^forte ball surface. HA was identified as adsorbing chemically to both BIOLOX^®^delta and BIOLOX^®^forte balls. Interestingly, Raman spectra of HA were found different for each type of ball. In addition, formation of a patterned film was observed on ceramic ball surfaces with HA.

All three model SFs did not exhibit considerable changes in Raman spectra between the before and the after-test samples. Especially Raman spectra of the healthy model SF were almost identical.

The chemisorption of the model SFs on the BIOLOX^®^delta ball was minimum which could be caused by chemical inertness of the zirconia-toughened alumina ball. Conversely, significant chemisorption films of osteoarthritic joint and total joint replacement model SFs were identified on the BIOLOX^®^forte ball surface. It is to be noted that the BIOLOX^®^forte ball is mainly alumina ceramic.

The coefficient of friction for BIOLOX^®^delta joints was significantly higher than for BIOLOX^®^forte joints with the presence of HA, the opposite was true in the presence of model SFs of healthy joint and of total joint replacement. However, the relationship between the friction coefficient and the chemisorbed films was not correlated for either ceramic joint.

## Figures and Tables

**Table 1:** Composition and concentration of the applied test lubricants.

Test Fluid	Albumin	*γ*-Globulin	Hyaluronic Acid	Phospholipids
	(mg/mL)	(mg/mL)	(mg/mL)	(mg/mL)
Albumin	28			
*γ*-globulin		11		
Hyaluronic Acid (HA)			2	
Healthy Joint (SF1)	20	3.6	2.5	0.15
After Total Joint Replacement (SF2)	26.3	8.2	0.87	0.35
Joint with Osteoarthritis (SF3)	24.9	6.1	1.49	0.34

## Data Availability

Data is contained within the article.

## Appendix A

The following tables summarize parameters of individual peaks shown in the Figure 2, Figure 3, Figure 4, Figure 5 and Figure 6. Given is the position of the peak and its FWHM, both in units cm^−1^.

**Table A1:** BIOLOX^®^delta and BIOLOX^®^forte ball surface with different protein fluids (Figure 2).

BIOLOX^®^delta ball surface with albumin	645 (21.6)	266 (21.0)
BIOLOX^®^delta ball surface with *γ*-Globulin	646 (21.6)	265 (24.2)
BIOLOX^®^delta ball surface	643 (21.6)	265 (24.2)
BIOLOX^®^forte ball surface with Albumin	646 (12.3)	418 (9.5)
BIOLOX^®^forte ball surface with *γ*-Globulin	646 (10.8)	418 (7.9)
BIOLOX^®^forte Ball Surface	645 (9.2)	418 (6.3)

**Table A2:** HA with BIOLOX^®^delta and BIOLOX^®^forte balls (Figure 3).

After test HA with BIOLOX^®^delta	993 (44.3)	1415 (23.8)	1080 (35)
After test HA with BIOLOX^®^forte ball	992 (19.2)	1409 (22.4)	1084 (27.8)
Blank HA	992 (17.7)	1416 (11.2)	1080 (21.9)

**Table A3:** BIOLOX^®^delta and BIOLOX^®^forte ball surface with HA (Figure 4).

BIOLOX^®^delta ball surface with HA	646 (27.8)	265 (21.0)
BIOLOX^®^delta Ball surface	643 (21.6)	265 (24.2)
BIOLOX^®^forte ball surface with HA	645 (13.9)	418 (9.5)
BIOLOX^®^forte Ball Surface	645 (9.2)	418 (6.3)

**Table A4:** Three types of model SFs with BIOLOX^®^delta and BIOLOX^®^forte ball (Figure 5).

After test SF1 with BIOLOX^®^delta ball	1654 (69.0)	1336 (41.8)	1003 (10.5)
After test SF1 with BIOLOX^®^forte ball	1656 (69.0)	1336 (43.5)	1003 (14.0)
Blank SF1	1651 (70.6)	1336 (41.9)	1003 (10.5)
After test SF2 with BIOLOX^®^delta ball	1652 (64.2)	1338 (41.9)	1003 (12.2)
After test SF2 with BIOLOX^®^forte ball	1656 (65.8)	1336 (45.2)	1003 (14.0)
Blank SF2	1656 (65.8)	1336 (40.2)	1003 (10.5)
After test SF3 with BIOLOX^®^delta ball	1654 (64.2)	1340 (41.9)	1003 (8.8)
After test SF3 with BIOLOX^®^forte ball	1657 (67.4)	1341 (60.4)	1003 (10.5)
Blank SF3	1656 (69.0)	1340 (43.5)	1004 (10.5)

**Table A5:** BIOLOX^®^delta and BIOLOX^®^forte ball surface with different types of model SFs (Figure 6).

BIOLOX^®^delta ball surface with SF1	644 (22.0)	457 (35.7)
BIOLOX^®^delta ball surface with SF2	644 (20.2)	476 (26.3)
BIOLOX^®^delta ball surface with SF3	644 (22.0)	476 (30.0)
BIOLOX^®^delta Ball surface	643 (21.6)	459 (37.8)
BIOLOX^®^forte ball surface with SF1	747 (19.9)	415 (13.2)
BIOLOX^®^forte ball surface with SF2	750 (16.3)	415 (9.5)
BIOLOX^®^forte ball surface with SF3	747 (18.1)	414 (11.3)
BIOLOX^®^forte Ball Surface	751 (13.7)	418 (6.3)
